# A plausible explanation of the b-value in the Gutenberg-Richter law from first Principles

**Published:** 2004-09-01

**Authors:** Panayiotis A. Varotsos, Nicholas V. Sarlis, Efthimios S. Skordas, Haruo Tanaka

**Affiliations:** *) Physics Department, University of Athens, Panepistimiopolis, Zografos 157 84, Athens, Greece; **) Earthquake Prediction Center, Tokai University

**Keywords:** Gutenberg-Richter relation, natural time, maximum entropy principle, universality

## Abstract

It is explained, from first Principles, why in the Gutenberg-Richter law (stating that the cumulative number of earthquakes N(>*M*) with magnitude greater than *M* is given by N(>*M*) ~ 10^−b^*^M^*) the so called b-value is usually found to be around unity varying only slightly from region to region. The explanation is achieved just by applying the analysis in the natural time domain, without using any adjustable parameter.

## Introduction

The best known scaling relation for earthquakes is the Gutenberg-Richter law (G-R).1) It states that the (cumulative) number of earthquakes with magnitude greater than *M* occurring in a specified area and time is given by

[1] $$\text{N}(>M)\sim 10^{-\text{b}M}$$

where b is a constant. Numerous publications have examined the spatial and temporal variations of the b-value, but it is currently considered 2) that it is generally a constant varying only slightly from region to region being in the range 0.8 ≤ b ≤ 1.2.

It has been recognized that G-R belongs to a broad range of natural phenomena that exhibit *fractal scaling*. 2),3) Such a scaling reflects that the number of earthquakes (occurring in a specified area and time) with rupture areas greater than *A* is given by 2),4)

[2] $$\text{N}(>A)=\text{C }A^{-\text{d}}$$

where d = D/2 and D stands for the fractal dimension. It has been suggested4) that Eqs. [1] and [2] are entirely equivalent with b = d = D/2 and hence the universal applicability of G-R implies universal fractal behavior of earthquakes.2)

The main aim of the present paper could be described as an attempt towards understanding the origin of the aforementioned constancy of the b- or D-value. Nowadays, it is generally accepted that main shocks can be considered in the frame of critical phenomena5) (e.g. see ref. ^5^) and references therein). Thus, keeping in mind that *criticality* is accompanied with *fractality*, we take here a power-law of the form of Eq. [2] or Eq. [1] as granted, and then attempt, from general principles, to identify from where this (almost) constancy of the b-value stems. Our procedure mainly consists of two basic steps: We first analyze the data obeying Eq. [1] in the frame of the newly introduced concept of natural time6)–14) and then investigate which b-value(s) results in maximizing the entropy. There have been many attempts to explain why the b-value is universally almost unity. However, to the authors’ knowledge, they are all model dependent and frequently use the concept of Self Organized Criticality (SOC).15) A usual line of thought include computational efforts related to well-known models of Burridge-Knopoff16) or Olami, Feder and Christensen17) that are focused on the mechanical phenomenology of earthquakes and actually capture essential elements of the genesis of a seismic event. In the frame of a single fault representation, the stick-slip mechanism is usually considered, in which the friction properties play the prominent role; we then additionally take into account that earthquakes occur in large areas characterized by a diversity of fault sizes and depths. Along these lines, the role of the geometry of the fault profiles, for example, in earthquake dynamics has been highlighted.18) Despite the several attempts that have been made towards G-R explanation, the most recent efforts 19) adopt the view that G-R has not yet been connected with general principles in Physics.

## The natural time

In a time series comprised of N events, the *natural time χ* *_k_* = *k*/*N* serves as an index6),7) for the occurrence of the *k*-th event. Concerning the analysis of seismicity, for example, the evolution of the pair (*χ* *_k_*, *M*_0_*_k_*) was considered,6),7),11),12) where *M*_0_*_k_* denotes the seismic moment of the *k*-th event, and the following continuous function *F*(*ω*) was introduced:

[3] $$F(\omega )=\sum_{k=1}^{N}M_{0k}\;\text{exp}\;\left(i\omega \frac{k}{N}\right)$$

where *ω* = 2***πφ***, and ***φ*** stands for the *natural frequency*. Normalizing *F*(*ω*):

[4] $$\Phi (\omega )=\frac{\sum_{k=1}^{N}M_{0k}\text{exp }\left(i\omega \frac{k}{N}\right)}{\sum_{k=1}^{N}M_{0k}}=\sum_{k=1}^{N}p_{k}\text{exp }\left(i\omega \frac{k}{N}\right)$$

where $p_{k}=M_{0k}/\sum_{k=1}^{N}M_{0k}$, we can define a kind of normalized power spectrum (or it may be more appropriately called energy spectrum) **Π**(*ω*):

[5] $$\Pi (\omega )={|\Phi (\omega )|}^{2}$$

It has been shown that, when the system enters the critical stage, the following relation holds:

[6] $$\Pi (\omega )=\frac{18}{5\omega^{2}}-\frac{6\text{cos}\omega }{5\omega^{2}}-\frac{12\text{sin}\omega }{5\omega^{3}}$$

We focus on the properties of **Φ**(*ω*) or **Φ**(***φ***) for natural frequencies ***φ*** close to zero. If we regard *p_k_* in Eq. [4] as a probability distribution, probability theory may lead to the analogy that, for the range of small ***φ***, **Π**(*ω*) or **Π**(***φ***) reduces to the characteristic function for *p_k_*. We may then be justified to further proceed with the probability theory, that the moments of a distribution and hence the distribution itself can be determined once the behaviour of the characteristic function of the distribution is known around zero. It has been argued11),13) that **Π**(***φ***), for ***φ*** → 0, can be considered as an order parameter and the corresponding probability density distribution, designated by *p*[**Π**(***φ***)] for ***φ*** → 0, is calculated by means of the procedure explained in the next section. Since, as mentioned above, we consider the earthquakes (main-shocks) in the frame of critical phenomena, we will use hereafter *p*[**Π**(***φ***)] for ***φ*** → 0 in the calculation of Entropy.

## The procedure followed and Results

We use here the Shannon information entropy,20) which is defined as $-\sum_{i}p_{i}\;\text{ln }p_{i}$. Shannon interpreted $-\sum_{i}p_{i}\;\text{ln }p_{i}$ (when considering *N* distinct events *A_i_*, 1 ≤ *i* ≤ *N*, pertaining to a model system; *p_i_* stands here for the probability for an event *A_i_* to occur in the game of chance with *N* possible outcomes) as a measure of missing information. This definition of the Shannon entropy $-\sum_{i}p_{i}\;\text{ln }p_{i}$ conveys a dual meaning of the uncertainty and information measure, which is usually seen as follows21): the less uncertainty of the system or its state, the larger (and more valuable) is the information we acquire as a result of the measurement upon the system and vice versa. Shannon entropy is static entropy and not a dynamic one.11),14) Static entropy solely depends on the probability distribution and hence remains unaltered when changing the order of the events, e.g., upon randomization (“shuffling”), while in a dynamic entropy the order of consecutive events plays an important role.

The Shannon entropy is used, because our interest here is focused on the statistical properties, while when studying the dynamic evolution of a system, the “entropy” in the natural time *S* = <*χ*ln*χ* > − <*χ* >ln<*χ* > should be preferred.14)

### The Maximum Entropy Principle

After more than 100 years, since the development of equilibrium statistical mechanics by the seminal work of Boltzmann and Gibbs, we still do not have a widely accepted formalism for non-equilibrium statistical mechanics. Gibbs procedure concerns with the equilibrium state only and consists of maximizing the quantity $-\sum_{i}p_{i}\;\text{ln }p_{i}$, with respect to the microstate probabilities *p_i_*, subject to the relevant constraints on the system. The maximization for the equal probabilities case, i.e., *p_i_* = *p*, results in the well known formula Boltzmann-Gibbs (BG) entropy, *S_BG_* = *k* ln *W*, where *W* = 1/*p* denotes the number of the accessible microstates. Since we deal here with non-equilibrium system, we rely on Jaynes formalism.22)–24) Jaynes,22),23) inspired from Shannon’s interpretation, suggested to look statistical mechanics as a form of *statistical inference* and to start statistical physics from a maximum entropy principle. Jaynes realized Gibbs’ formalism of equilibrium systems as just one example of a general form of statistical inference (“MaxEnt”) which could be extended to non-equilibrium systems. His hypothesis that the phase-space paths adopted by non-equilibrium systems (cf. paths rather than states are the central objects of interest in non-equilibrium systems) are distributed according to MaxEnt. This has been found successful in several cases,25) including the recovery23) of the results of linear transport theory (Onsager, Kubo and others) and SOC.26) In MaxEnt, we compute $-\sum_{\Gamma }p(\Gamma )\text{ln }p(\Gamma )$ where ***Γ*** represents an entire path through phase space, spanning the duration of the non-equilibrium experiments in question.

### Details of our procedure and the results obtained

**Fig. 1:**
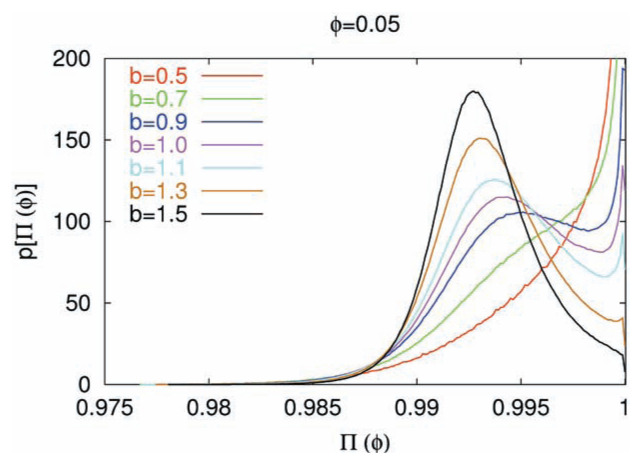
The probability density function of **Π**(***φ*** = 0.05) in the natural time-domain. *p*[**Π**(***φ*** = 0.05)] versus **Π**(***φ*** = 0.05) for several values of b. Note how the feature of the curve changes significantly upon increasing b from b = 0.5 to b = 1.5.

The following steps have been applied. We first generated, for each b-value, artificial data comprised from 500,000 events, that obey Eq. [1] above a certain magnitude threshold (e.g., *M* ≥ 0). This was repeated for various b-values by keeping the total number (500,000) of events constant. The data for b~1 should be equivalent to the “shuffled”14) data of an actual earthquake catalogue and their probability density functions (pdf) should be the same. (As it is explained further in the Discussion, we focus here on the self-similarity exponent that stems from the distribution of the process’ increments only, not from the memory of the process. Note that natural time domain analysis can serve two purposes, the study of a dynamic evolution as well as a statistical study by investigating the original time series and the shuffled one, respectively). The data were subsequently analyzed in the natural time domain, for each b-value, with the following procedure6),8),11),12): First, calculation of the power spectra **Π**(***φ***) was made for small ***φ*** -values, e.g., ***φ*** = 0.05, since we are interested in the case of ***φ*** → 0, for the reasons explained in the second section, for an event taking time windows for 6 to 40 consecutive events. This process was performed for all the events by scanning the whole dataset via a Monte Carlo procedure. As an example, we plot in Fig. 1, the quantity *p*[**Π**(***φ***)] versus **Π**(***φ***) for ***φ*** = 0.05 for several b-values. Recall, that this figure may be regarded as depicting the probability distribution of the order parameter versus the value of the order parameter. Since the fluctuations of the order parameter become larger as we approach a critical point,14) we now study the Shannon Information Entropy associated with *p*[**Π**(***φ***)] for ***φ*** =0.05 (a measure of these fluctuations), which is expected to maximize close to the critical point. In such a frame, we do not make any use of the MaxEnt Principle. An alternative frame, which does make use of MaxEnt in the Jaynes formalism, is the following: In a slowly driven system (e.g., that is frequently adopted in the study of self-organized criticality), we investigate the same number of events above a threshold — that obey power law energy distribution — and examine which power law exponent corresponds to the maximum entropy, i.e., the most likely behavior in nature.

**Fig. 2:**
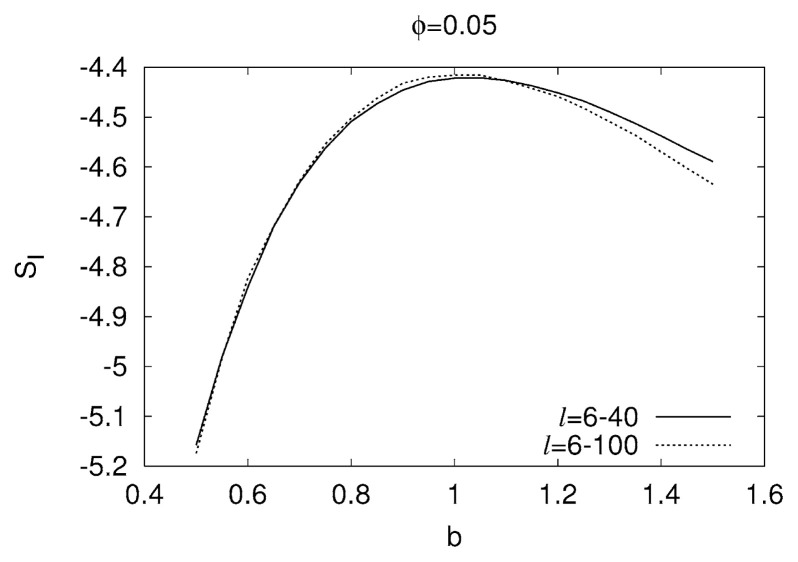
The calculated values of the differential entropy versus the exponent b. The location of the maximum (around b ≈ 1) does not depend on the time-window length *l* used in the calculation: Solid line: *l* = 6–40; dotted: *l* = 6–100 consecutive events.

In order to have a better accuracy, we compute, for each b-value studied, the Shannon information entropy of the continuous probability distribution *p*[**Π**(***φ***)] for ***φ*** = 0.05, i.e., *S_I_* = − *∫p*[**Π**(***φ***)]ln*p*[**Π**(***φ***)]*d***Π**(***φ***) which is usually termed *differential entropy* (e.g., see ref. 21) and references therein) and it can attain negative values also since the pdf may become larger than unity. Finally, we investigate the resulting *S_I_*-values versus b. Such a plot is given in Fig. 2 in which the *S_I_*-values are depicted versus the corresponding b-values. An inspection of this figure reveals that *S_I_* maximizes at a value of b around unity in agreement with the experimental values. We emphasize that the location of this maximum at b~1 is not practically affected if the time-window length (*l*) chosen is increased from *l* = 6–40 to *l* = 6–100 as shown by the two lines in Fig. 2. Even if *l* = 1000, its value decreases only slightly, i.e., b~0.95. Since in the experimental studies for the determination of the b-value, earthquake populations of the order ~10^2^–10^3^ events are usually taken into account, we consider here as satisfactory the agreement between the experimental b-values and the one(s) obtained on theoretical grounds. A study at larger *l*-values is still in progress, and the results will be shortly reported elsewhere.

## Discussion

In the present paper, we focused our attention on the magnitude (moment) distribution of the seismic events, without paying any attention to the memory that may be present in an actual time series of seismic events. However, we emphasize that, in principle, two different origins of self-similarity in stationary time-series11),27) can be distinguished: (1) the so-called long memory and (2) the distribution of the process’ increments. Since our procedure has been solely focused on the second origin in the present paper (because we studied “shuffled” time series), we briefly discuss below how one can distinguish which of the two origins is responsible for the self-similarity observed.

We first comment on Hurst analysis, which results in the so-called Hurst exponent *H_H_*. We emphasize that, unfortunately, *H_H_* is not an estimator27) of the self-similarity index even though it is so commonly thought. This exponent gives only information on the correlations in the time series measured at different time scales, and hence reveals the memory of the investigated process (e.g., when 1/2 < *H_H_* < 1 the time series is called persistent and it has a long-memory property, while when *H_H_* = 1/2 the changes in the values of a time series are random). The memory, however, is not the only possible origin of self-similarity,27) because a second origin of self-similarity comes from the process’ increments distribution. In order to make clear this point, let us consider a process which has purely random increments with infinite variance28); this process can be self-similar with index of self-similarity different from 1/2. An example of such a process is27) the Levy stable motion with stationary, independent and identically distributed (i.i.d.) increments with symmetric *α* -stable distribution. The Hurst analysis of that process gives *H_H_* = 1/2, which just shows the lack of memory, while the actual self similarity exponent *H* may lie for example between 0.8 and 1.0.27) In other words, the widely used procedures of Hurst analysis (exponent *H_H_*) and Detrended Fluctuation analysis (exponent *α ^DFA^*) give us information on memory only, and not on the distribution of the process’ increments.

Hence, in order to investigate correctly the self-similarity property, one should distinguish between the long-memory property and the process’ increments distribution properties. Along these lines, the (random) “shuffling” of the original data provides a useful tool: If the self-similarity stems from the process memory only, then the exponents of the shuffled data are different from those in the original data and changed to *H_H_* = *α _DFA_* = 1/2 since the shuffling of the original data destroys the correlations and the resulting time series is without memory.14) On the other hand, if the self-similarity stems only from the process’ increments infinite variance, then the exponents of the original data do not change, i.e., they are equal to those in the shuffled data. In the general case, i.e., if the self-similarity stems from both origins, i.e., memory and increments’ distribution, we observe a partial change of the exponents *H_H_* and *α _DFA_*. Thus, it becomes clear why in our procedure here, the *p*[**Π**(***φ***)] calculation was made on the shuffled data, since we were solely interested on the increments’ distribution.

We now briefly discuss, in simple words, some additional reasoning that led us to identify **Π**(***φ***) (***φ*** → 0) as an order parameter. We started with the model,29) that a Seismic Electric Signals (SES) activity is emitted when the stress (control parameter) in the focal area approaches a critical stress ***σ***_cr_. Analysing the seismicity in a candidate area, which can be estimated from the observed SES data, the following interesting property has been shown6),8): If we set the natural time for the seismicity zero at the initiation time of the concerned SES-activity, we can form time series of seismic events in natural time for various time windows as the number of consecutive earthquakes *N* increases. When we compute **Π**(***φ***) for each of the time windows, we found that, in the range 0 < ***φ*** ≤ 0.5, it approaches, as *N* increases from 6 to some value less than 40, to that given by Eq. [6]. This equation has also been found to describe in a universal way all the SES activities observed to date.7) Interestingly, this approach of **Π**(***φ***) of seismicity to that of SES activity happens only a few days before the anticipated main shock. Such a behavior has been checked to occur just before all strong earthquakes (*M* ≥ 5.8) in Greece since 1988. In other words, **Π**(***φ***) abruptly increases from the value obeying Eq. [6] to the value **Π**(***φ***) = 1, since the energy — and hence the *M*_0_ value — of the main shock exceeds by orders of magnitude the *M*_0_ values of the preceding small earthquakes. (This is reminiscent of the case in which — under an external field — the magnetization, which is a usual example of an order parameter, abruptly changes from zero to a non-zero value upon reaching the critical point). This behaviour observed in Greece, is likely to be the case for the main shocks in Japan and San Andreas Fault in view of the following fact12) when sliding a window of the same length, i.e., *l* = 6–40 consecutive events through the corresponding seismic catalogues, we found that the most probable value of **Π**(***φ***) (***φ*** → 0) is the one obeying Eq. [6].

Finally, we point out that the procedure followed in this paper is quite general. It shows that the natural time domain analysis,6),7) if we consider **Π**(***φ***) (***φ*** → 0) as an order parameter, implies that data obeying Eq. [1] — or Eq. [2] — *should* exhibit b-values around unity. In other words, it seems that the almost constant value b~1 (for *l* ≤ 10^3^) is just a consequence of the physical expectation that the information entropy *S_I_* associated with *p*[**Π**(***φ***)] for small ***φ*** (i.e., the probability distribution of the order parameter) should become maximum. The extent to which the experimental results in diverse fields verify this conclusion is currently explored.

The following clarification should be added. As explained in the previous section, the main aspect from which our present procedure stems, is the following: The fluctuations of order parameter are expected to maximize close to the critical point. The Shannon information entropy associated with *p*[**Π**(***φ***)] for small ***φ*** was just used here as the most appropriate measure of the order parameter fluctuations. Our present study refers, of course, to a non-equilibrium critical system, but we have already verified that the same idea holds for equilibrium systems as well. In particular, our simulations for the 2D and 3D Ising models do show that the Shannon entropy associated with the corresponding probability distribution of the magnetization maximizes close to the critical temperature. Details on these results will be shortly published elsewhere.

## Conclusion

The natural time-domain analysis reveals that the exponent b in the Gutenberg-Richter (G-R) law, i.e., N(>*M*) ~ 10^−b^*^M^*, should be around unity as actually observed. This conclusion is drawn without using *any* adjustable parameter.
